# A Decline in p38 MAPK Signaling Underlies Immunosenescence in *Caenorhabditis elegans*

**DOI:** 10.1371/journal.pgen.1002082

**Published:** 2011-05-19

**Authors:** Matthew J. Youngman, Zoë N. Rogers, Dennis H. Kim

**Affiliations:** Department of Biology, Massachusetts Institute of Technology, Cambridge, Massachusetts, United States of America; Stanford University Medical Center, United States of America

## Abstract

The decline in immune function with aging, known as immunosenescence, has been implicated in evolutionarily diverse species, but the underlying molecular mechanisms are not understood. During aging in *Caenorhabditis elegans*, intestinal tissue deterioration and the increased intestinal proliferation of bacteria are observed, but how innate immunity changes during *C. elegans* aging has not been defined. Here we show that *C. elegans* exhibits increased susceptibility to bacterial infection with age, and we establish that aging is associated with a decline in the activity of the conserved PMK-1 p38 mitogen-activated protein kinase pathway, which regulates innate immunity in *C. elegans*. Our data define the phenomenon of innate immunosenescence in *C. elegans* in terms of the age-dependent dynamics of the PMK-1 innate immune signaling pathway, and they suggest that a cycle of intestinal tissue aging, immunosenescence, and bacterial proliferation leads to death in aging *C. elegans*.

## Author Summary

Aging is associated with a diminished capacity to resist infection in evolutionarily diverse species including humans, mice, and fruit flies. Few studies have addressed the underlying genetic and molecular basis of this phenomenon. Here we examined immune function during aging in the nematode *Caenorhabditis elegans*, which we demonstrate exhibits a progressive age-dependent increase in susceptibility to the human pathogen *Pseudomonas aeruginosa*. Our studies suggest that, during aging in *C. elegans*, the activity of the PMK-1 p38 mitogen-activated protein kinase pathway, a conserved pathway involved in pathogen defense, sharply declines. We propose a model in which age-related damage to intestinal tissue impairs immune function and therefore promotes infection, which in turn amplifies tissue damage, thus setting in motion a self-perpetuating cycle that gradually erodes host defense.

## Introduction

Aging is associated with increased mortality from infection in evolutionarily diverse species [1]. These observations have been attributed in part to an age-associated decline in immune function, termed immunosenescence. In vertebrates, a marked decrease in T cell receptor diversity associated with thymic involution during aging is a major contributor to the immunosenescence of the adaptive immune system [2]. Age-related changes of the innate immune system and their influence on susceptibility to infectious diseases are less well understood. Dysfunction of innate immune function later in life is associated with “inflammo-aging,” a phenomenon in which an aberrant increase in the production of pro-inflammatory cytokines can contribute to tissue damage [3]. Such dysregulation may promote disease, as suggested in a recent report indicating that increased inflammatory signaling through Toll Like Receptor 3 contributes to increased pathology in West Nile Virus infection in the elderly [4]. Changes in the amplitude of immune signaling activity during aging have also been observed in invertebrates. For example, in aging *Drosophila* basal immune signaling is constitutively increased, yet immune responses induced by bacteria appear to be attenuated [5], and older flies are more susceptible to infection [6].

Studies of aging in *Caenorhabditis elegans* have revealed several biomarkers associated with aging, including increased intestinal proliferation of the relatively non-pathogenic *Escherichia coli* strains that are used as food sources during assays of animal longevity. Intact bacterial cells are rarely detected within the intestinal lumen of younger animals, but ultrastructural analysis of aging *C. elegans* has revealed discrete areas of bacterial packing and local catastrophic plasma membrane disruption events, along with extensive deterioration of intestinal tissues [7], [8]. There is evidence to suggest that the accumulation of *E. coli* within the intestinal lumen during aging is a cause of death in older animals because *C. elegans* propagated on killed or non-dividing *E. coli* live longer than animals propagated on live *E. coli* [7], [9]. These data suggest that bacterial pathogenesis is a major contributor to aging and mortality in *C. elegans* and raise the possibility of an age-dependent decline in immune function during aging.

Innate immunity in *C. elegans* is regulated by a conserved PMK-1 p38 mitogen-activated protein kinase (MAPK) pathway [10] that is required for resistance to a diverse range of pathogenic bacteria and fungi [10], [11]. PMK-1 regulates the transcription factor ATF-7, which activates intestinal expression of genes encoding proteins that contribute to host defense such as C-type lectins, lysozymes, and putative antimicrobial peptides [12]–[14]. While PMK-1 is critical for immune protection during larval development and early adulthood, its role in innate immunity during aging has not yet been investigated. In this paper, we report the results of genetic, gene expression profiling, and biochemical studies during aging in *C. elegans* which together demonstrate a marked decline in PMK-1 later in life. We suggest a model that involves a cycle of immunosenescence, increased bacterial infection and proliferation, and progressive intestinal tissue deterioration that accelerates mortality in aging *C. elegans*.

## Results

### *C. elegans* resistance to bacterial infection declines with advancing age

**Figure 1:**
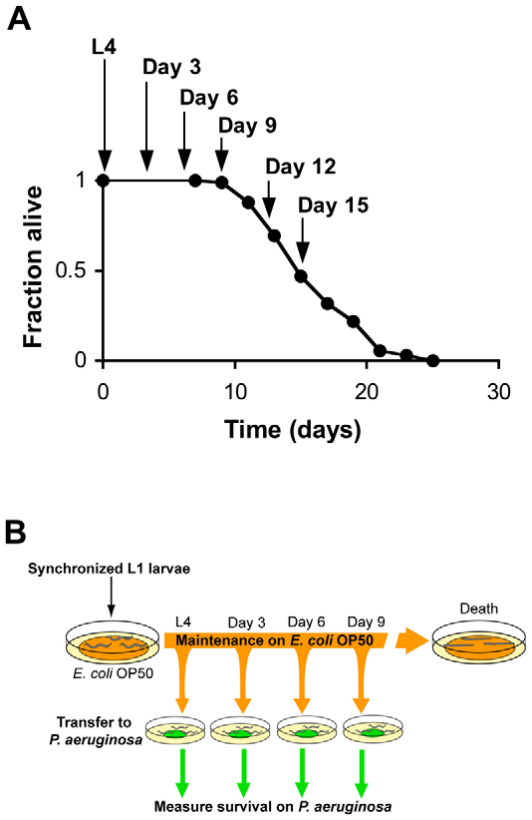
Systematic analysis of the survival of aging *C. elegans* upon challenge with pathogenic bacteria. (A) Survival of wild type strain N2 maintained on *E. coli* OP50 plotted as fraction of worms alive versus time. Arrows indicate ages of worms when *P. aeruginosa* infection was initiated, when reporter gene expression was examined, and/or when total RNA was harvested for microarray analysis. (B) Schematic of *P. aeruginosa* infection assay. At the indicated ages, subsets of worms from a synchronized population cultured on a lawn of *E. coli* OP50 were transferred to plates containing a lawn of *P. aeruginosa* PA14, and their survival was monitored twice daily thereafter.

**Figure 2:**
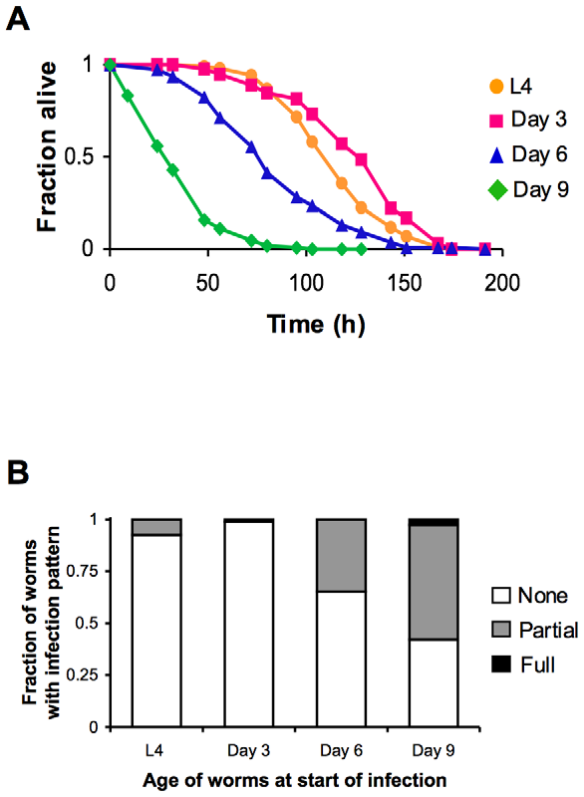
Increased susceptibility of *C. elegans* to lethal infection with aging. (A) Survival of wild type strain N2 transferred from *E. coli* OP50 to *P. aeruginosa* PA14 at L4 (orange), Day 3 (magenta), Day 6 (blue), or Day 9 (green) plotted as fraction of worms alive versus time. (B) Accumulation of *P. aeruginosa* within the intestinal lumen of *C. elegans* at ∼24 h post-infection. Wild type N2 strain late larval stage (L4) and adult worms were infected with a strain of *P. aeruginosa* that expresses GFP and then scored the next day according to the extent of bacterial colonization of the intestine. The pattern of *P. aeruginosa* infection in individual animals was classified as either “None” when no GFP-expressing *P. aeruginosa* could be detected in the intestine, “Partial” when *P. aeruginosa* colonization of the intestine was incomplete or was localized to a bolus, or “Full” when the intestinal lumen was completely packed with bacteria along its entire length.

We began our study of the dynamics of innate immunity during aging by investigating the age-dependent variation in susceptibility of the *C. elegans* laboratory wild type strain N2 to *Pseudomonas aeruginosa* PA14, a human opportunistic pathogen that also kills *C. elegans* by an infection-like process in the intestine [15]. The lifespan of *C. elegans* wild type strain N2 propagated on the relatively non-pathogenic bacterial strain *E. coli* OP50 is approximately 21 days (Figure 1A). To determine how aging influences the susceptibility of *C. elegans* to infection, we challenged *C. elegans* with *P. aeruginosa* at the L4 larval stage (1 day pre-adulthood), and at Day 3, Day 6, and Day 9 of adulthood by transferring them from standard plates seeded with *E. coli* OP50 to plates seeded with *P. aeruginosa* PA14 (Figure 1B). Pathogen susceptibility was not assayed beyond this period because mortality begins to be observed after Day 12 of adulthood in *C. elegans* propagated on *E. coli* OP50 (Figure 1A). We observed a decline in the survival time of *C. elegans* adults transferred to *P. aeruginosa* with advancing age (Figure 2A). Our data are consistent with prior observations that aging *C. elegans* have diminished survival upon challenge with pathogenic bacteria [15]–[17] and establish that *C. elegans* exhibit a progressive age-dependent increase in susceptibility to infection.

The age-dependent increase in susceptibility of *C. elegans* to killing by *P. aeruginosa* suggests older animals have diminished protective responses to pathogenic bacteria, which may manifest as an accelerated accumulation of bacteria during infection. To test this possibility, L4 larval stage animals and Day 3, 6, and 9 adults were challenged with a strain of *P. aeruginosa* which expresses GFP. After exposure to GFP-expressing *P. aeruginosa* PA14, *C. elegans* were scored for the degree of bacterial accumulation within the intestinal lumen. We found that one day after an infection was initiated, over half of Day 9 adult *C. elegans* accumulate *P. aeruginosa* within at least a portion of their intestine, and that the intestine of some worms is completely filled with bacteria (Figure 2B). After the same duration of exposure to *P. aeruginosa*, 30% of Day 6 adults and less than 10% of Day 3 adults had accumulated detectable levels of GFP-expressing *P. aeruginosa* in their intestines. The rate of accumulation of *P. aeruginosa* within the intestine increases in an age-dependent manner and parallels the increased susceptibility to infection (Figure 2A), suggestive of a decline in immune function during aging.

### A decline in PMK-1 activity with intestinal tissue aging

**Figure 3:**
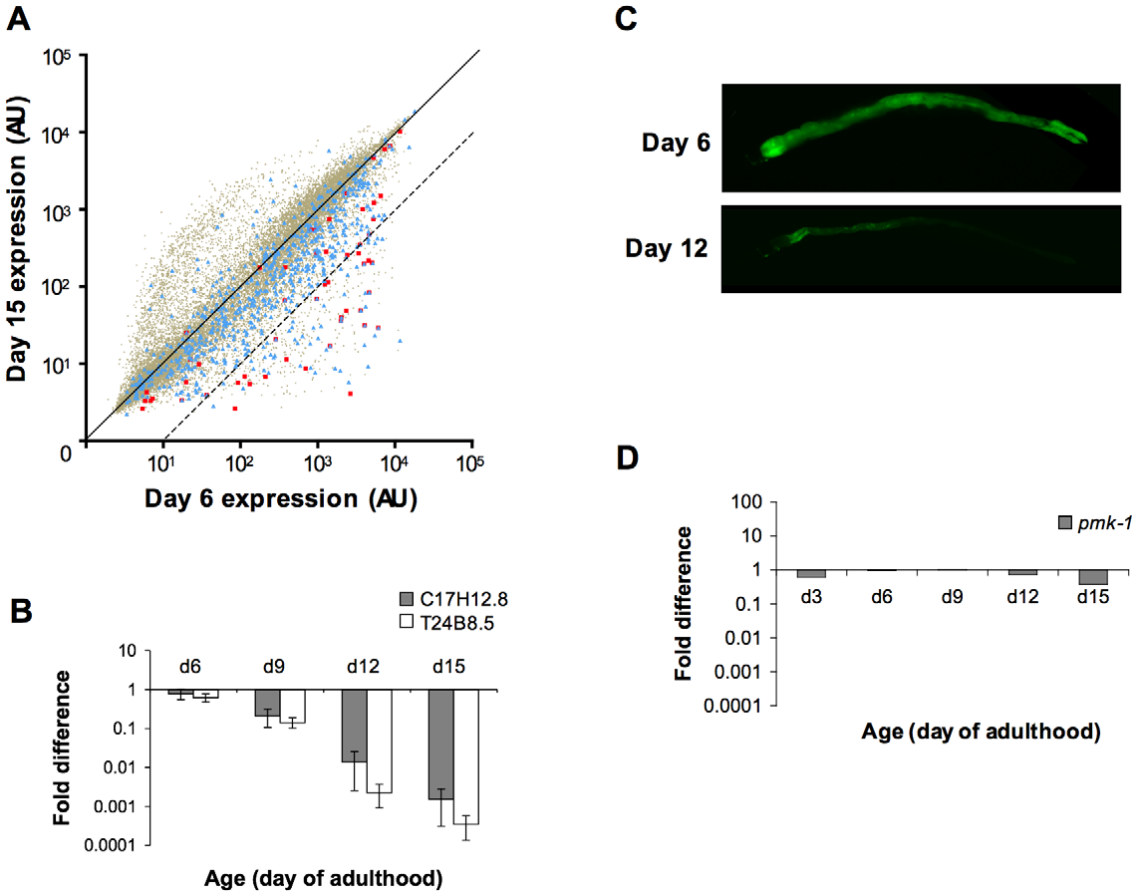
A decline in expression of PMK-1 transcriptional targets during aging in the *C. elegans* intestine. (A) Scatter plots comparing gene expression levels in the *C. elegans* wild type N2 strain at Day 15 versus Day 6 of adulthood. Each dot represents an individual gene; brown, all genes on the full-genome microarray; blue, genes previously shown to exhibit enriched expression levels in the *C. elegans* intestine; red, genes previously identified as being regulated by the PMK-1 pathway. Genes on the solid diagonal line are expressed at equivalent levels at both time points. Genes below the dashed diagonal line are downregulated by more than 10-fold between Day 6 and Day 15 of adulthood. (B) qRT-PCR of endogenous C17H12.8 and T24B8.5 mRNA levels during aging relative to expression at Day 3 of adulthood. The average of three experiments using independent biological replicates of total RNA for cDNA synthesis is shown. Bars indicate standard deviation. (C) Fluorescence microscopy of *C. elegans* carrying the *agIs219* transgene, a GFP reporter of PMK-1 activity, at the indicated ages. (D) qRT-PCR of *pmk-1* transcript levels during aging relative to expression at L4. The average of two experiments using independent biological replicates of total RNA for cDNA synthesis is shown.

We sought to identify the genetic determinants underlying the phenomenon of increasing susceptibility to infection with advancing age. Previously, microarray-based gene expression studies of aging *C. elegans* revealed numerous changes in gene expression during the aging process [18], [19]. Upon comparing these data with our previously reported microarray-based identification of PMK-1 p38 MAPK pathway transcriptional targets [13], we observed an enrichment for genes regulated by the PMK-1 p38 MAPK pathway among genes downregulated with aging. To explore this observation further and to identify genes downregulated during mid-to-late adulthood, we carried out a full-genome microarray analysis of aging *C. elegans*, analyzing gene expression levels in synchronized populations of N2 worms at Day 6 of adulthood and Day 15 of adulthood (Figure 3A). We found that when compared with expression levels at Day 6 of adulthood, approximately 12% of genes in the *C. elegans* genome (2535 genes) are downregulated by 2-fold or more by Day 15 (Figure 3A and Table 1). From this set of genes, we identified 379 that were downregulated by 10-fold or greater in Day 15 adults compared to expression levels in Day 6 adults (Table S1). To determine whether this subset of genes included those which are important for conferring resistance to infection, we asked if genes previously shown to be upregulated during an infection in young adult *C. elegans* were in our list and found a 7-fold enrichment (*p* = 1.2×10^−21^) for genes induced by *P. aeruginosa* infection [13]. Moreover, several significant Gene Ontology (GO) terms associated with genes induced by pathogen exposure [14], [20], [21] were found among the genes that are most downregulated during mid-to-late adulthood, including “peptidase activity,” “hydrolase activity,” and “lipid metabolic activity” (Table S2).

**Table 1:** Distribution of intestine-enriched genes and PMK-1 transcriptional targets among genes downregulated at Day 15 versus Day 6 of adulthood.

		Day 15/Day 6 Expression Level		
Genes	Number of Genes	≥2-fold Decrease	≥10-fold Decrease	*p*-value
Whole genome	∼20,000	2535/20,000	379/20,000	
Intestine-enriched	659	305/659	60/659	2.0×10^−24^
PMK-1 targets	58	42/58	26/58	8.7×10^−30^

The number of intestine-expressed genes (based on a list compiled from previous studies [24], [25]) and PMK-1 transcriptional targets (identified previously [13]) with levels of expression reduced by either ≥2-fold or ≥10-fold in Day 15 animals compared to expression levels in Day 6 adults is reported. The statistical significance of the fold difference among genes downregulated by 10-fold or more between Day 6 and Day 15 of adulthood is represented by a hypergeometric *p*-value.

GO analysis of genes downregulated between Days 6 and 15 of adulthood in *C. elegans* revealed an enrichment for some of the same GO terms associated with the transcriptional targets of both PMK-1 and DAF-16, a conserved FOXO transcription factor that, in the absence of inhibition through the insulin signaling pathway, upregulates the expression of genes involved in lifespan determination, stress response, and innate immunity [13], [22], [23] (Table S2). We examined the expression levels of downstream targets of PMK-1 and DAF-16 among genes that are downregulated during aging. When mapped onto a scatter plot comparing gene expression in Day 15 adult *C. elegans* to expression levels in Day 6 adults, almost all PMK-1 targets show sharp downregulation (Figure 3A). DAF-16 targets, however, exhibit a pattern that is more representative of the genome-wide changes in expression that occur during aging. Specifically, while the expression levels of many DAF-16 targets either remain unchanged or decrease, other target genes appear to be upregulated during mid- to late adulthood, suggesting that DAF-16 remains active later in life, in contrast to the apparent age-dependent decline in PMK-1 activity (Figure S1).

Of the genes that are downregulated by at least 2-fold in older adult animals, 42 have previously been shown to be regulated by the PMK-1 p38 MAPK pathway [13]. A total of 26 of these PMK-1 transcriptional targets are among the 379 genes that are sharply downregulated late in aging, which represents a dramatic and significant 24-fold enrichment (*p* = 8.7×10^−30^) for PMK-1-regulated genes among genes exhibiting markedly decreased expression levels between Day 6 and Day 15 of adulthood. This enrichment is particularly evident when highlighting PMK-1-regulated genes on a scatter plot of expression levels of Day 15 versus Day 6 adults (Figure 3A). Quantitation of endogenous transcript levels of two PMK-1 targets in wild type *C. elegans* during mid- to late adulthood by qRT-PCR confirmed that a gradual decline in the expression of PMK-1-regulated genes indeed occurs between Day 6 and Day 15 of adulthood and is most pronounced after Day 9 (Figure 3B). Furthermore, this analysis corroborated the magnitudes of the changes that were measured by microarray-based expression profiling. Supporting these measurements of endogenous PMK-1 target transcript levels, we also observed diminished expression of the *agIs219* reporter transgene, which consists of the promoter for a transcriptional target of PMK-1, T24B8.5, fused to GFP [12], in the intestinal cells of older adult *C. elegans* (Figure 3C). In sum, these studies indicate a striking age-dependent decline in the expression of PMK-1 transcriptional targets.

Electron microscopy of aging *C. elegans* has revealed dramatic deterioration of multiple tissues, including the intestine, with advancing age [8]. We anticipated that tissue deterioration would result in the global attenuation of the expression of intestinal genes in older adult animals. We compared the gene expression profile for genes differentially expressed at Day 15 versus Day 6 of adulthood with genes previously defined as being expressed in the *C. elegans* intestine [24], [25]. Consistent with the possibility that tissue deterioration causes a global decline in intestinal cell transcription toward the end of life, we found that over 40% of intestine-expressed genes (305/659) are among the genes that are downregulated by at least 2-fold late in aging (Figure 3A and Table 1). A total of 60 of these genes were among the 379 genes with a 10-fold or greater decrease in expression in Day 15 adults compared to Day 6 animals (Figure 3A and Table 1). This is greater than the number of intestine-expressed genes expected to be found by chance and represents a 5-fold enrichment (*p* = 2.0×10^−24^) for intestine-expressed genes among those that are the most downregulated in older adult animals.

**Figure 4:**
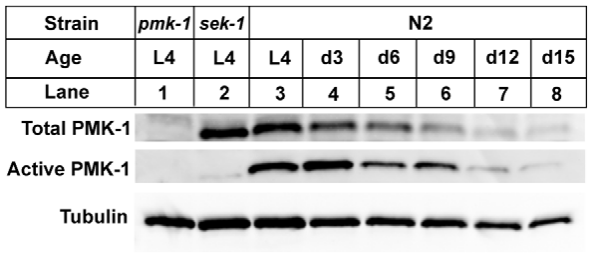
A gradual reduction in the levels of total and activated PMK-1 protein throughout adulthood in *C. elegans*. Immunoblot analysis of total and activated PMK-1 during aging. Total protein isolated from L4 larval stage *pmk-1(km25)* (lane 1), L4 *sek-1(km4)* (lane 2) or L4 and adult wild type N2 strain (lanes 3-8) was separated by SDS-PAGE, and immunoblots were decorated with antibodies to either PMK-1 (Total PMK-1), phosphorylated PMK-1 (Active PMK-1) or β-tubulin (Tubulin).

The sharp decline in the expression of transcriptional targets of the PMK-1 pathway with advancing age, even relative to the age-related decrease in intestinal gene expression, suggests that the activity of the PMK-1 pathway fades in late adulthood in *C. elegans*. We asked whether *pmk-1* itself might be regulated at either the level of mRNA, protein, or phosphorylation during aging. By qRT-PCR analysis, we observed that compared to expression levels at the L4 larval stage, *pmk-1* expression showed a relatively modest decline of no more than two-fold in 12- and 15-day-old worms, far less than the degree to which PMK-1 transcriptional target expression was reduced during aging (Figure 3D). In contrast, the levels of PMK-1 protein showed a marked decline starting around Day 9 of adulthood, to the point where by Day 15, <16% of the levels of PMK-1 observed in late larval stage animals remained (Figure 4). Using an antibody that specifically recognizes the doubly phosphorylated activated form of PMK-1, we found a corresponding age-dependent decrease in the levels of activated PMK-1 during aging. Our studies of *pmk-1* transcript and protein levels during aging suggest that diminishing PMK-1 protein abundance is responsible for the attenuated activity of the PMK-1 signaling pathway later in life.

**Figure 5:**
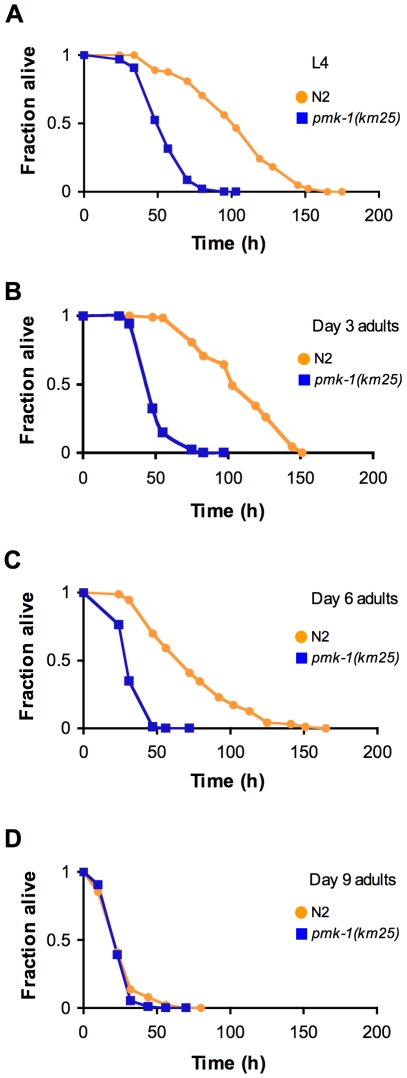
Age-dependent decrease in the contribution of PMK-1 p38 MAPK signaling to *C. elegans* immunity. (A) Survival of wild type strain N2 (orange) and the *pmk-1*(*km25)* mutant (blue) transferred from *E. coli* OP50 to *P. aeruginosa* PA14 at L4, and at Days 3, 6, and 9 of adulthood (B–D, respectively), plotted as fraction of worms alive versus time.

Previously, we established that mutations that attenuate the activity of the PMK-1 pathway result in enhanced susceptibility to killing by *P. aeruginosa* during larval development and young adulthood [10]. The striking decrease in expression of PMK-1-regulated genes and the observed decline in PMK-1 levels in aging animals suggested a diminished role for the PMK-1 pathway in host defense towards the end of life. Consistent with this prediction, we observed that with advancing age, the difference in survival between wild type and *pmk-1* mutants challenged with *P. aeruginosa* subsides (Figure 5A–5D). Whereas *pmk-1* loss of function shortens the LT_50_ (median time to death) of 3- and 6-day-old adult *C. elegans* challenged with *P. aeruginosa* by 50% and ∼60% respectively, when infection is initiated at Day 9 of adulthood, wild type animals and *pmk-1* mutants die at an equivalent rate.

Considering the decrease in abundance of the active, phosphorylated form of PMK-1 during aging (Figure 4) we tested whether inactivating VHP-1, a phosphatase which negatively regulates PMK-1 during larval development in *C. elegans* [26], could rescue the enhanced susceptibility of older adult animals to infection.

We reasoned that reduced VHP-1 function might prevent or delay the decrease in PMK-1 activity that occurs during aging. RNAi-mediated knock-down of *vhp-1* during adulthood failed to improve the resistance of Day 9 adults to *P. aeruginosa* infection (Figure S2). These data provide further evidence to suggest that the age-dependent decline in PMK-1 activity later in life results from the waning abundance of PMK-1 protein and not from a decrease in PMK-1 phosphorylation.

PMK-1 mediates host defense in *C. elegans* by regulating the expression of genes encoding proteins important for the response to infection [13], [14]. In earlier studies, we found that RNAi of individual transcriptional targets of the PMK-1 pathway did not yield a pathogen susceptibility phenotype, indicative of functional redundancy among putative immune effectors regulated by PMK-1 [13]. We repeated this analysis in older *C. elegans*, carrying out the inactivation of 29 individual PMK-1 targets by RNAi to determine if we could phenocopy the susceptibility phenotype of Day 6 *pmk-1* mutants upon infection with *P. aeruginosa* (Figure S3, Figure S4). Included among these genes were several found to be upregulated in response to *P. aeruginosa* infection [13]. For 27 of 29 targets, RNAi-mediated gene inactivationn produced no reproducible effect on pathogen susceptibility. A modest effect on pathogen susceptibility was observed upon RNAi of *nlp-31* (B0213.6) and *tag-38* (B0222.4), but the small effects suggest that downstream targets of PMK-1 primarily function redundantly to mediate innate immunity in aging *C. elegans*, as was observed for young adult *C. elegans* [13].

**Figure 6:**
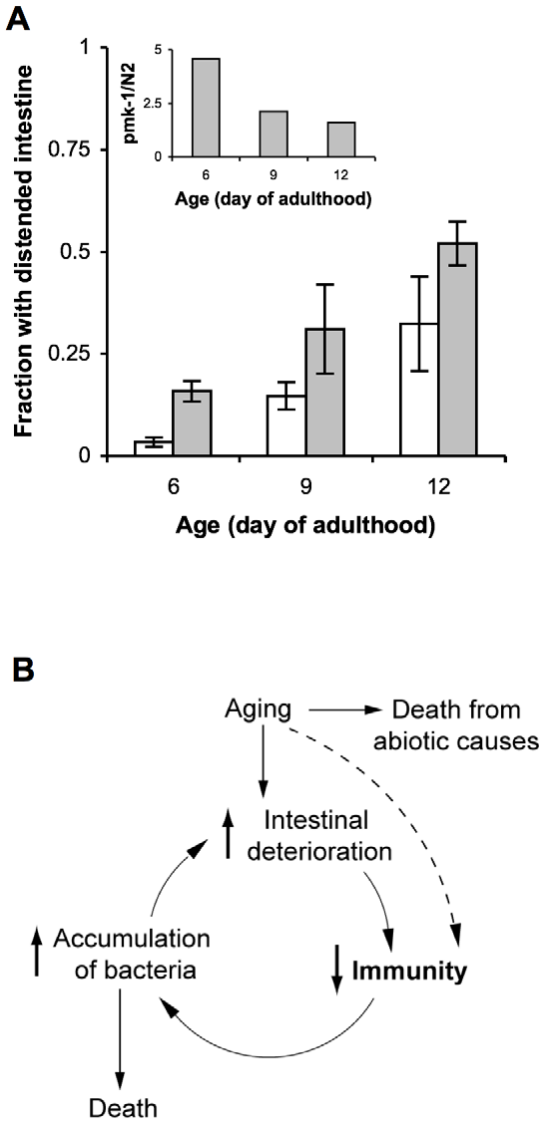
A cycle of intestinal tissue aging, immunosenescence, and progressive intestinal proliferation of bacteria towards the end of life in *C. elegans*. (A) Fraction of wild type N2 *C. elegans* (white bars) or *pmk-1(km25)* mutants (grey bars) maintained on lawns of *E. coli* OP50 that exhibit intestinal distention at Days 6, 9, and 12 of adulthood. Inset, ratio of the average number of *pmk-1* mutants with intestinal distention versus the average number of wild type animals with intestinal distention at Days 6, 9, or 12 of adulthood. (B) As the primary immune cells in *C. elegans*, age-related damage to intestinal cells may impair their ability to execute processes required for immune protection, such as the expression of immune effector proteins, including those regulated by the PMK-1 pathway. The consequent reduction in host defense would lead to increased colonization of the *C. elegans* intestine by pathogenic microbes, which contribute to and amplify intestinal cell deterioration during the infection process. Thus a self-perpetuating cycle of increased intestinal deterioration, decreased immunity, and increased accumulation of bacteria may underlie immunosenescence and significantly contribute to mortality later in life.

The standard laboratory food source for *C. elegans*, *E. coli* OP50, is likely pathogenic to aging animals [7], [9], [27]. Therefore, if the role of PMK-1 in host defense is to protect against infection, then the effects of exposure to *E. coli* that mimic an infection should be enhanced by the progressive decline in PMK-1 activity during aging. Intestinal distention due to bacterial packing develops in aging adults [7], with ultrastructural evidence that this accumulation of bacteria can contribute to death [8]. Consistent with the role of PMK-1 in innate immunity, we observed that the *pmk-1* mutant exhibited increased accumulation of *E. coli* with aging relative to wild-type *C. elegans* (Figure 6A). As assessed by intestinal distention, we observed a 5-fold greater prevalence of *E. coli* accumulation within the intestine of *pmk-1* mutants as compared to wild type *C. elegans* at Day 6 of adulthood. Whereas the accumulation of *E. coli* increased in an age-dependent manner, we observed that the difference between wild type and *pmk-1* mutant *C. elegans* diminished with advancing age (Figure 6A). By Day 9 and Day 12 of adulthood, twice as many *pmk-1* mutants exhibited a distended intestinal lumen as compared to age-matched wild type animals, in contrast to the 5-fold difference observed at Day 6 of adulthood. These data corroborate our observations that the contribution of PMK-1 to host defense declines in an age-dependent manner during adulthood.

## Discussion

### Immunosenescence in *C. elegans*

Although a number of observations in diverse species suggest dysfunction of the innate immune system with aging, how innate immunity changes over the course of the aging process has been unclear. Our data suggest that the diminishing contribution of the PMK-1 pathway to resistance to *P. aeruginosa* infection in aging animals underlies the increasing susceptibility to infection of *C. elegans* with advancing age. The global decline in intestinal gene expression with advancing age is consistent with ultrastructural observations that show a deterioration of intestinal cells with aging. Because the intestinal cells are the principal cells of host defense in *C. elegans*, functioning as a mucosal defense barrier, tissue aging may compromise the ability of these cells to function in resistance to infection. Thus, we hypothesize that the aging of intestinal cells may contribute to immunosenescence which, in turn, as we discuss below, may promote the aging process.

### A cycle of intestinal tissue aging, immunosenescence, and bacterial infection promotes aging and mortality in *C. elegans*

While the activity of the genetic determinants of aging appears to be coordinated among the various tissue types in *C. elegans* [28]–[31], different tissues exhibit distinct degrees of deterioration with advancing age. For example, whereas neurons exhibit minimal evidence of age-related damage in *C. elegans*, intestinal cells exhibit morphological changes during aging, including areas of plasma membrane disruption and the disappearance of microvilli [8]. Stochastic localized aging has also been reported in murine models where mucosal immunity in the gastrointestinal tract may exhibit early aging relative to components of systemic immunity such as the spleen [32]. Unlike the intestinal cells of vertebrates, intestinal cells of *C. elegans* are post-mitotic, and thus bacteria present in the intestinal lumen may accelerate tissue aging during the infection process through the secretion of toxins and other virulence mechanisms. Therefore, investigating the interaction between older adult *C. elegans* and pathogenic bacteria that infect the gut provides a system for understanding the chronic effects of aging on immune function and, in a reciprocal manner, the contribution of innate immunity to tissue aging.

The proliferation of bacteria in the intestinal lumen of aging *C. elegans* suggests two opposing hypotheses regarding innate immune function. One possibility is that increased bacterial packing and proliferation might lead to the pathological activation of innate immune responses that may directly contribute to host lethality. Derangement and hyperactivation of innate immunity is observed in septic shock during overwhelming infection in humans [33]. In this scenario the sustained, excessive activity of the immune system could result in collateral tissue damage that exacerbates age-related deterioration. Indeed, we recently characterized how the innate immune response itself can be lethal during *C. elegans* development in the absence of homeostatic endoplasmic reticulum stress responses [34]. However, we found no evidence of increased immune function in older adult *C. elegans*. Instead our data suggest an alternative situation in which innate immune function declines during aging, likely contributing to progressive bacterial proliferation. Infection has been hypothesized to be a major cause of death in aging worms, based on lifespan extensions conferred by feeding animals killed instead of live bacteria [7], [9] and the aforementioned ultrastructural observations [8]. Because the intestinal cells function as immune cells in *C. elegans*, intestinal tissue deterioration may impair mucosal immune defenses. Taken together with prior observations of intestinal tissue aging and bacterial proliferation in aging animals, our data on *C. elegans* immunosenescence suggest a downward spiral during aging such that a decline in immunity promotes the increased proliferation of bacteria, which in turn, may accelerate intestinal tissue aging and deterioration (Figure 6B). Ultimately, the senescence of innate immunity suggests that aging *C. elegans* lack components of host defense that confer protection from infection earlier in life, allowing for the proliferation of even relatively non-pathogenic bacteria that may substantially contribute to mortality. We speculate that immunosenescence may herald the transition to the terminal stages of the aging process in *C. elegans*.

## Materials and Methods

### *C. elegans* growth for aging and lifespan assays

Animals were synchronized by hypochlorite treatment and L1 arrest. Starved L1 larvae were placed onto NGM plates seeded with *E. coli* OP50 and grown at 20°C to the L4 stage. L4 animals were transferred by chunking to 10 cm NGM plates supplemented with 50 µg/ml 5-fluorordeoxyuridine (FUdR) and seeded with *E. coli* OP50. Worms were maintained on these plates at 20°C until they were used for experiments at Days 3, 6, 9, 12, or 15 of adulthood. Plates that became contaminated or significantly depleted of *E. coli* OP50 over time were discarded.

To determine the lifespan of *C. elegans* wild type strain N2, worms were synchronized and grown to L4 as described above and then picked over to 6 cm *E. coli* OP50-seeded NGM plates containing 50 µg/ml FUdR at 20°C where they were maintained throughout the assay. A total of approximately 100 worms were transferred to three NGM plates (∼30 worms/plate) in each replicate assay. Worms were scored every 2–4 days beginning at Day 7 of adulthood by gently prodding with a platinum wire to test for touch sensitivity as an indication of life or death. Lifespan is defined as the time elapsed from when worms were transferred to FUdR plates (time  = 0 on survival curves) to when they were scored as dead.

### Analysis of bacterial accumulation

To analyze bacterial accumulation in the intestinal lumen during infection with *P. aeruginosa*, worms were transferred from plates containing *E. coli* OP50 to plates containing a strain of *P. aeruginosa* PA14 which expresses GFP [15]. Animals were monitored for the presence of GFP-*P. aeruginosa* within the intestinal lumen by fluorescence microscopy ∼24 h after initiating the infection. At the time of scoring, in order to visualize worms in the absence of interfering GFP signal from the bacterial lawn, worms were transferred from plates containing GFP-*P. aeruginosa* to *E. coli* OP50 plates. At 4x magnification bacterial accumulation was scored according to the following criteria: “none” if no GFP signal could be detected in the intestinal lumen, “partial” if GFP-*P. aeruginosa* was observed in only a portion of the intestine or was dispersedly distributed throughout the intestine, or “full” if a robust GFP signal could be detected without interruption along the entire length of the intestinal lumen.

To measure the prevalence of *E. coli* accumulation within the intestinal lumen during aging, wild type N2 *C. elegans* or *pmk-1* mutants were maintained on lawns of *E. coli* OP50 at 20°C as described above. Beginning at Day 6 and continuing at regular intervals until Day 12 of adulthood, animals were examined for evidence of bacterial packing as manifest in intestinal distention. At each time point, at least 70 worms of each strain were anaesthetized with 10 mM sodium azide and mounted on glass slides for examination by Nomarski microscopy. At 100x magnification animals were scored positive for intestinal distention if sections of their intestinal lumen appeared to be abnormally wide and resulted in the apparent compression of the bordering intestinal cells.

### Isolation of RNA for quantitative RT-PCR and microarray analysis

Synchronized populations of *C. elegans* were generated as described above and then harvested at the indicated ages by rinsing plates with M9 buffer and collecting animals into 15 ml conical tubes. After allowing worms to settle, the supernatant was removed and animals were rinsed with fresh M9. Washed worms were transferred to a screw-capped tube, resuspended in Tri reagent (Ambion) and vortexed before flash freezing in liquid nitrogen for storage at −80°C. Following phenol-chloroform extraction, RNA was precipitated in isopropanol and resuspended in RNAse-free water. For replicate microarray experiments and qRT-PCR, RNA was isolated from three populations of N2 worms that were independently propagated.

### Quantitative RT-PCR (qRT-PCR) analysis

Total RNA was reverse-transcribed using the Retroscript kit (Ambion). The resulting cDNA was used as the template in triplicate qRT-PCR reactions using SYBR Green detection (Roche) in a Mastercycler Realplex (Eppendorf). Primers for amplification of T24B8.5 and C17H12.8 were as described in a previous study [13]. The ΔΔC_t_ method was used determine relative mRNA levels using expression of *tba-1* (primers described previously [35]) as a normalization control. The abundance of *tba-1* did not change during aging (data not shown) when normalized to the levels of either *nhr-23* (primers described previously [13]) or *act-1* (primers described previously [36]).

### RNA hybridization and analysis of microarray chips

RNA was quantified and quality confirmed using an Agilent 2100 Bioanalyzer (Agilent Technologies, Inc.). 100 ng of total RNA was amplified and labeled using the NuGEN Ovation RNA amplification v2 kit (NuGEN Technologies, Inc.) according to the manufacturer's instructions. Samples were hybridized to GeneChip *C. elegans* genome microarrays at 45°C for 16 h, and chips were scanned using an Affymetrix GeneChip Scanner 3000 7G (Affymetrix Inc.). Absent/present calls were generated by analyzing the data with Microarray Suite version 5 (MAS5.0). Processed arrays were normalized and log_2_ expression values output using GCRMA [37]. Microarray data have been deposited in NCBI's Gene Expression Omnibus and are accessible online through GEO Series accession number GSE21784 (http://www.ncbi.nlm.nih.gov/geo/query/acc.cgi?acc=GSE21784).

### Statistical analysis of microarray data

The GCRMA output was filtered such that genes without at least one present call were eliminated. For the remaining genes, log_2_ expression values were normalized by first calculating the mean of the expression values of all replicates at all time points for an individual gene and then subtracting the result from every expression value corresponding to that gene in all of the replicates. To determine age-dependent changes in gene expression the mean log ratio of gene expression at two different points during aging was calculated, and a 2-tailed unpaired Student's *t* test was used to identify genes with statistically significant (*p*-value ≤0.05) changes in expression levels as a function of age.

Lists of genes with significant changes in expression during aging were queried for the presence of transcriptional targets of PMK-1 or for genes with enriched levels of expression in the *C. elegans* intestine. PMK-1 targets were identified in a previous study [13] and are defined as genes upregulated by 2.5-fold or greater in *glp-4(bn2)* versus *glp-4 (bn2)*; *sek-1(ag1)* with a *p*-value of <0.05 (t-test). A list of genes with enriched intestinal expression was compiled by combining the unique results from SAGE library analysis of intestinal tissue isolated from adult *C. elegans* [24] and from microarray-based studies of intestine-expressed genes in L4 larval stage animals [25]. In cases where no CDS identifier was available in the WormBase genome database (http://www.wormbase.org), the gene was excluded from comparison to our microarray data.

To determine the fold enrichment of PMK-1 target genes, PA14-induced, or intestine-expressed genes in our data sets, the fraction of target genes in a given data set (e.g. 26 PMK-1-regulated genes/379 genes with decreased expression at Day 15 versus Day 6) was divided by the fraction of target genes present in the *C. elegans* genome (e.g. 58 total PMK-1 targets/20,000 genes in the genome). *p*-values for fold enrichment were determined by calculating hypergeometric distribution. Significant GO ontology terms associated with subsets of genes in the microarray experiments were identified using the web-based application FatiGO (http://babelomics3.bioinfo.cipf.es/) [38]. Scatter plots were constructed by plotting average raw transformed intensities from three independent biological replicates for each gene on the GeneChip *C. elegans* genome microarrays using GraphPad Prism (version 4.0b).

### Immunoblot analysis

To prepare total protein lysates for western analysis, worms were harvested as described above for RNA isolation, except that after transferring animals to a screw-capped tube, the supernatant was replaced with 300 µl of SDS Sample Buffer (4% sodium dodecyl sulfate [SDS], 100 mM Tris·Cl pH 6.8, 20% glycerol). After boiling worms for 15 min, insoluble material was pelleted by centrifuging tubes at 10,000 g for 5 min, and the supernatant was transferred to a new tube which was immediately frozen at −80°C for storage until further analysis. The amount of total protein present in the lysates was determined using a BCA Protein Assay kit (Pierce). To prepare samples for SDS-PAGE, 100 mM dithiothreitol (DTT) and 0.1% bromphenol blue were added before boiling for 5 min. Proteins were separated on 10% SDS-PAGE gels (Bio-Rad) and then transferred to nitrocellulose membranes (Bio-Rad) according to the manufacturer's instructions. For western blotting analysis, membranes were probed with anti-PMK-1 antibody ([10]; gift of K. Matsumoto), anti-phospho-p38 (Promega) or anti-β-tubulin (E7, Developmental Studies Hybridoma Bank, Iowa City, Iowa). Immune complexes were detected using HRP-conjugated secondary antibodies (GE Healthcare) followed by chemiluminescence (Amersham ECL Advance Western Blotting Detection Kit, GE Healthcare). Western blots were quantitated using ImageJ 1.43u software (available online at http://rsb.info.nih.gov/ij/download.html).

### RNAi of PMK-1 transcriptional targets, *vhp-1*, and *pmk-1*

RNAi by bacterial feeding using *E. coli* HT115 bearing plasmids corresponding to the transcriptional targets of PMK-1 (obtained from the Ahringer library [39]), *vhp-1* [26] or *pmk-1* [26] was carried out as described [40] with the following modifications. No tetracycline or isopropyl β-D-1 thiogalactopyranoside (IPTG) was added to liquid bacterial cultures. RNAi plates contained a final concentration of 2 mM IPTG to induce expression of RNAi constructs and were not supplemented with tetracycline. For experiments involving RNAi treatment of adult animals, 50 µg/ml FUdR was added to the plates. In experiments involving RNAi of PMK-1 targets, synchronized *C. elegans* L1 larvae were added to RNAi bacteria plates, then transferred to new RNAi plates containing FUdR once they reached the L4 stage where they were maintained until challenged with *P. aeruginosa* at Day 6 of adulthood. For RNAi of *vhp-1*, Day 6 adult *C. elegans* were transferred from *E. coli* OP50 to RNAi food where they were maintained until challenged with *P. aeruginosa* at Day 9 of adulthood.

## Supporting Information

Figure S1 DAF-16 targets are representative of genome-wide changes in expression during aging in *C. elegans*. Scatter plot comparing gene expression levels in the *C. elegans* wild type N2 strain at Day 15 versus Day 6 of adulthood. Each dot represents an individual gene; brown, all genes on the full-genome microarray; red, genes previously identified as being regulated by the DAF-16 pathway [13], [22], [23]. Genes on the solid diagonal line are expressed at equivalent levels at both time points.
(TIF)

Figure S2 Inactivation of VHP-1 during adulthood fails to rescue age-related enhanced susceptibility to pathogen in *C. elegans*. Wild type N2 animals were treated with RNAi directed against *vhp-1* (orange), *pmk-1* (black) or an empty vector (L4440, blue) from Day 6 until Day 9 of adulthood. Survival of RNAi-treated animals transferred to *P. aeruginosa* PA14 at Day 9 of adulthood is plotted as fraction of worms alive versus time.
(TIF)

Figure S3 Functional redundancy among PMK-1 transcriptional targets in *C. elegans* innate immunity (Replicate 1). (A–G) Wild type N2 *C. elegans* were treated with RNAi directed against the indicated PMK-1 transcriptional targets or with empty vector L4440 from the L1 stage until Day 6 of adulthood. Survival of RNAi-treated animals transferred to *P. aeruginosa* PA14 at Day 6 of adulthood is plotted as fraction of worms alive versus time. The results of the first biological replicate are shown.
(TIF)

Figure S4 Functional redundancy among PMK-1 transcriptional targets in *C. elegans* innate immunity (Replicate 2). (A–G) Wild type N2 *C. elegans* were treated with RNAi directed against the indicated PMK-1 transcriptional targets or with empty vector L4440 from the L1 stage until Day 6 of adulthood. Survival of RNAi-treated animals transferred to *P. aeruginosa* PA14 at Day 6 of adulthood is plotted as fraction of worms alive versus time. The results of the second biological replicate are shown.
(TIF)

Table S1 PMK-1 targets downregulated by 10-fold or more in Day 15 adults, relative to expression levels in Day 6 adults. List of PMK-1 targets identified amonggenes with a ≥10-fold reduction in expression levels in Day 15 adults compared to Day 6 expression levels (*p*≤0.05).
(XLS)

Table S2 Significant GO terms associated with genes downregulated by 10-fold or more between Day 6 and Day 15 of adulthood in *C. elegans*. List of Gene Ontology (GO) terms describing biological processes or molecular functions with significant enrichment (*p*<0.05) among genes downregulated during aging in *C. elegans*. For comparison, significant GO terms (*p*<0.05) associated with transcriptional targets of PMK-1 and DAF-16 are also included.
(XLS)
