# Supplementary figures and images for: A Decline in p38 MAPK Signaling Underlies Immunosenescence in Caenorhabditis elegans
Source: PLoS Genet. 2011 May 19;7(5):e1002082. doi: 10.1371/journal.pgen.1002082 (PMC3098197; doi:10.1371/journal.pgen.1002082)

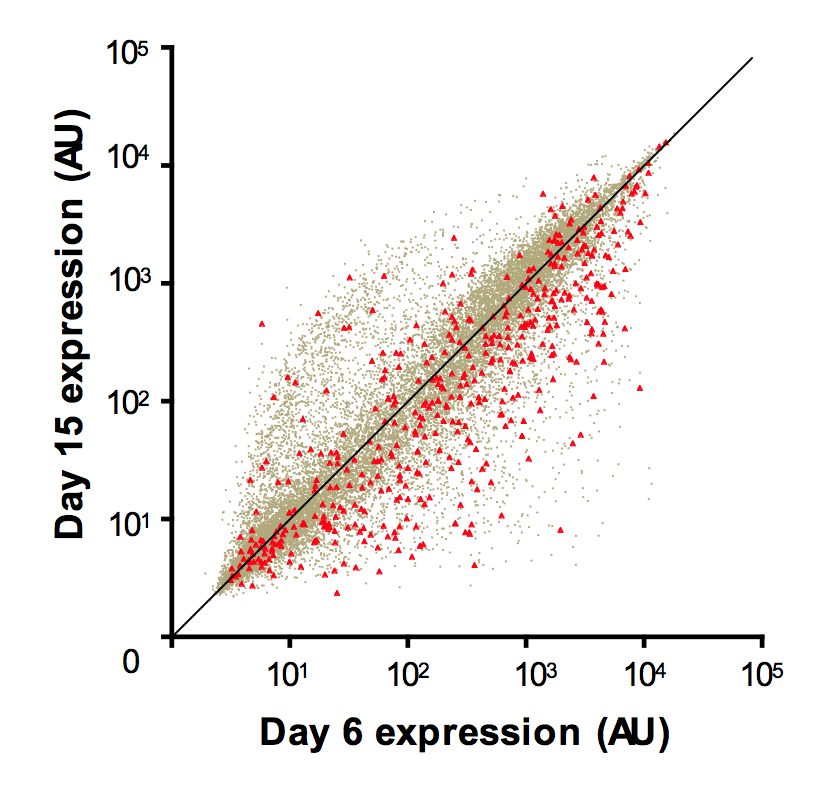

Supplement: Figure S1 — DAF-16 targets are representative of genome-wide changes in expression during aging in C. elegans. Scatter plot comparing gene expression levels in the C. elegans wild type N2 strain at Day 15 versus Day 6 of adulthood. Each dot represents an individual gene; brown, all genes on the full-genome microarray; red, genes previously identified as being regulated by the DAF-16 pathway [13], [22], [23]. Genes on the solid diagonal line are expressed at equivalent levels at both time points. (TIF) [file pgen.1002082.s001.tif]

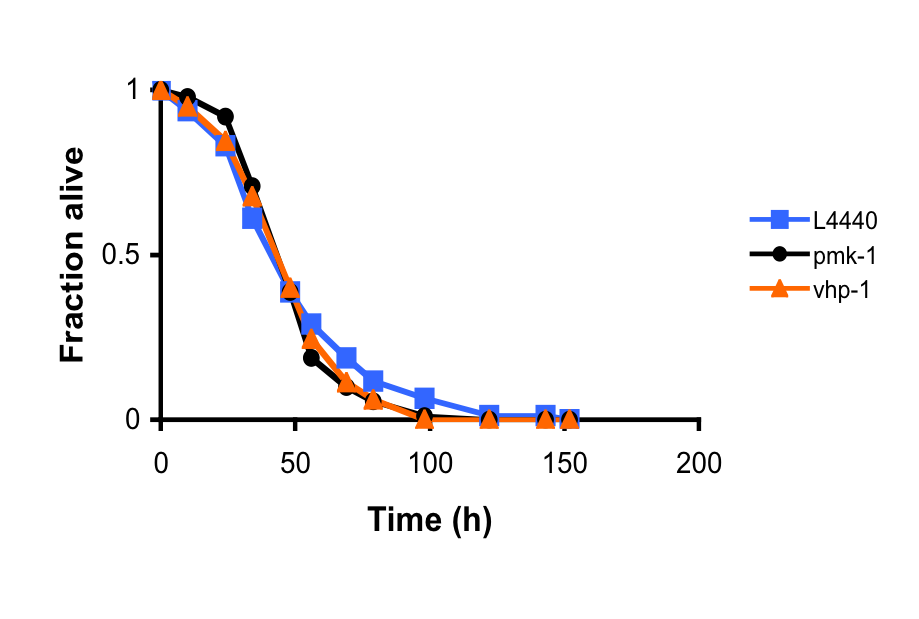

Supplement: Figure S2 — Inactivation of VHP-1 during adulthood fails to rescue age-related enhanced susceptibility to pathogen in C. elegans. Wild type N2 animals were treated with RNAi directed against vhp-1 (orange), pmk-1 (black) or an empty vector (L4440, blue) from Day 6 until Day 9 of adulthood. Survival of RNAi-treated animals transferred to P. aeruginosa PA14 at Day 9 of adulthood is plotted as fraction of worms alive versus time. (TIF) [file pgen.1002082.s002.tif]

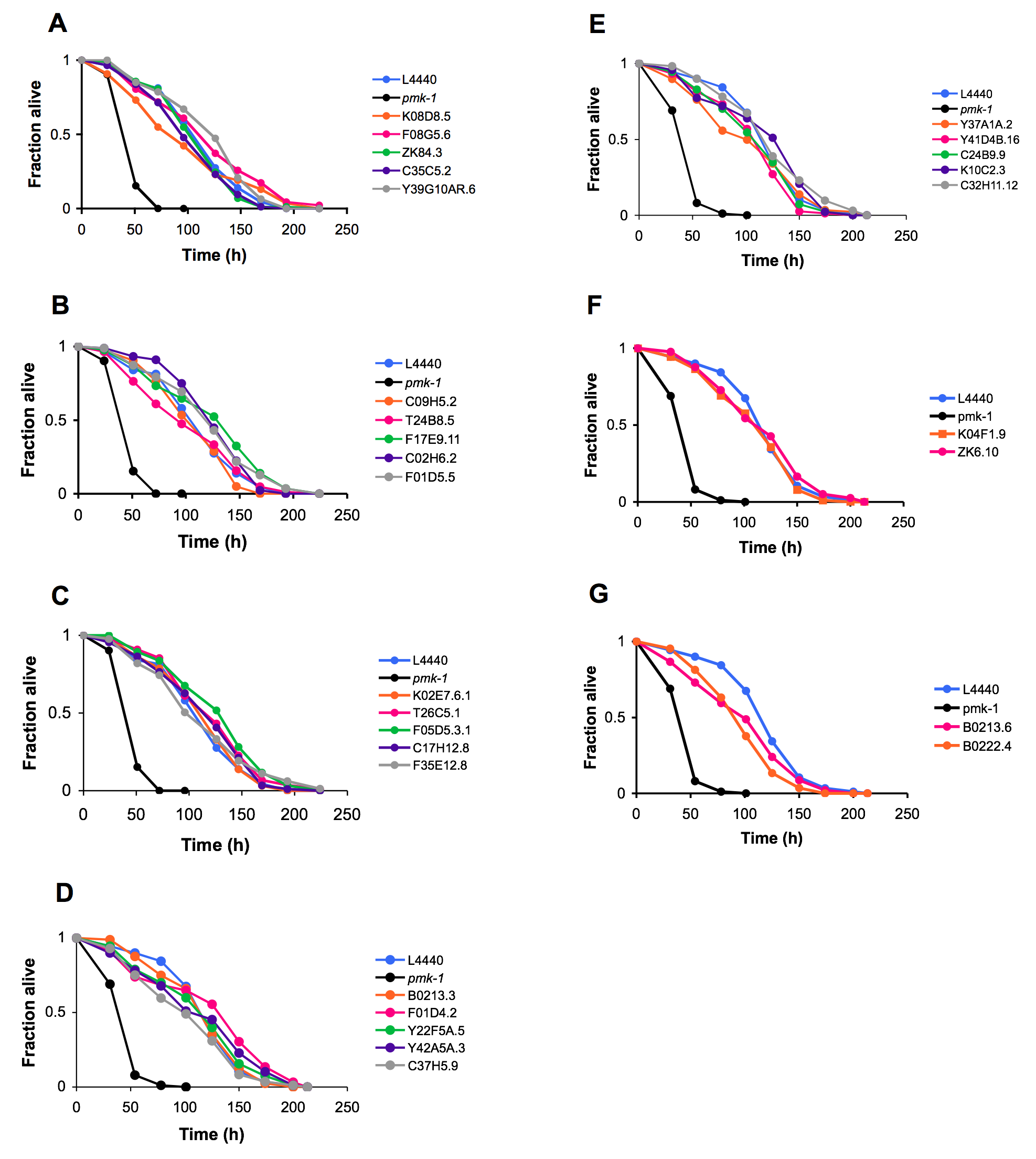

Supplement: Figure S3 — Functional redundancy among PMK-1 transcriptional targets in C. elegans innate immunity (Replicate 1). (A–G) Wild type N2 C. elegans were treated with RNAi directed against the indicated PMK-1 transcriptional targets or with empty vector L4440 from the L1 stage until Day 6 of adulthood. Survival of RNAi-treated animals transferred to P. aeruginosa PA14 at Day 6 of adulthood is plotted as fraction of worms alive versus time. The results of the first biological replicate are shown. (TIF) [file pgen.1002082.s003.tif]

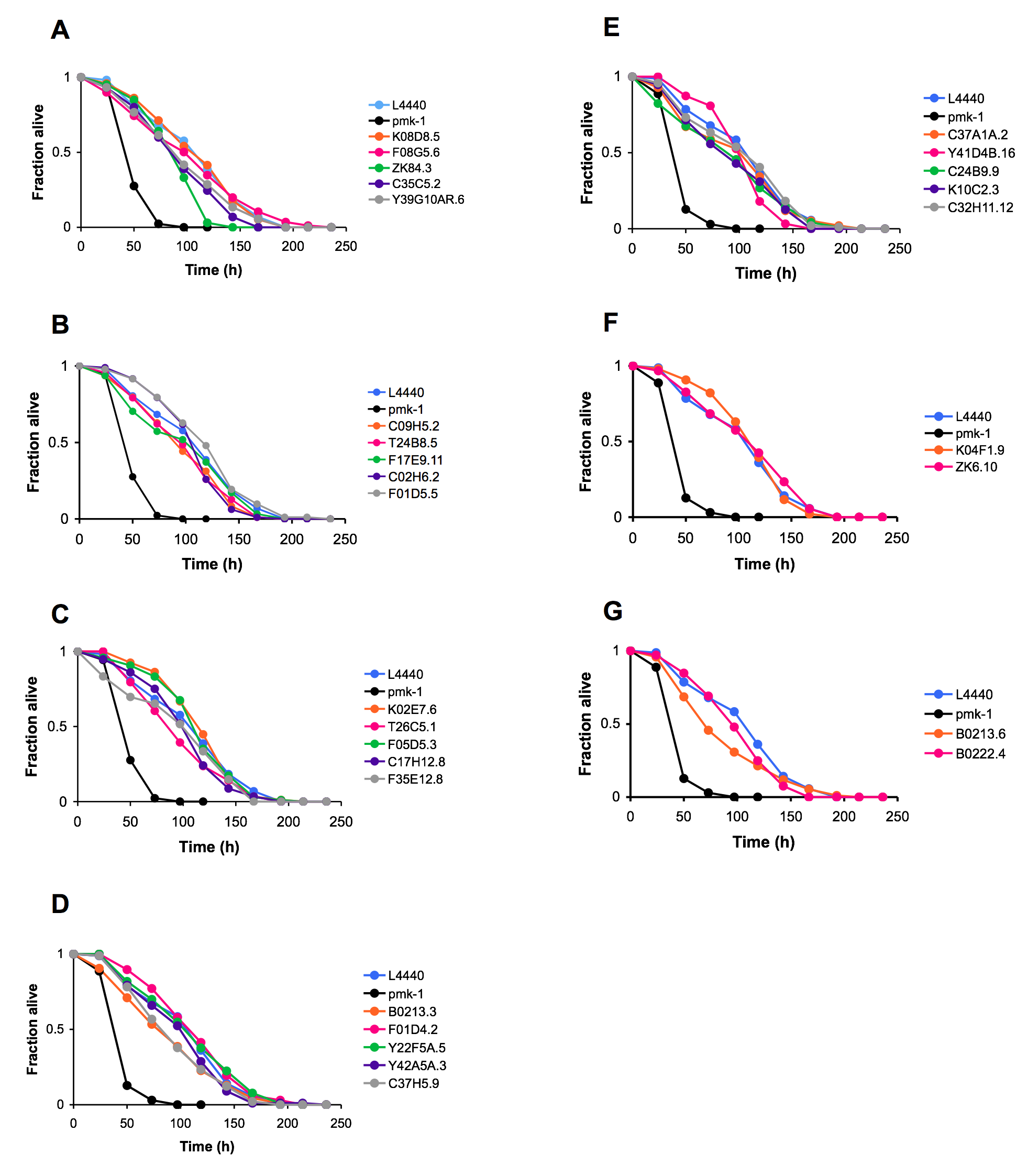

Supplement: Figure S4 — Functional redundancy among PMK-1 transcriptional targets in C. elegans innate immunity (Replicate 2). (A–G) Wild type N2 C. elegans were treated with RNAi directed against the indicated PMK-1 transcriptional targets or with empty vector L4440 from the L1 stage until Day 6 of adulthood. Survival of RNAi-treated animals transferred to P. aeruginosa PA14 at Day 6 of adulthood is plotted as fraction of worms alive versus time. The results of the second biological replicate are shown. (TIF) [file pgen.1002082.s004.tif]
